# Comparison of the McGrath video laryngoscope and macintosh direct laryngoscope in obstetric patients: A randomized controlled trial

**DOI:** 10.12669/pjms.35.2.646

**Published:** 2019

**Authors:** Melike Korkmaz Toker, Basak Altıparmak, Ayse Gul Karabay

**Affiliations:** 1 *Melike Korkmaz Toker, Mugla Sitki Kocman University Research and Training Hospital, Anesthesiology and Reanimation Department, Mugla, Turkey*; 2 *Basak Altiparmak, Mugla Sıtkı Kocman University, Department of Anesthesiology and Reanimation, Mugla, Turkey*; 3 *Ayse Gul Karabay, Ota-Jine Med Private Hospital, Anesthesiology Clinic, Istanbul, Turkey*

**Keywords:** Cesarean section, Direct Laryngoscopy, General anesthesia, McGrath Video laryngoscope

## Abstract

**Objective:**

In obstetric patients’ airway, guidelines have recommended the availability of advanced airway equipment. Our aim was to compare the larynx visualization provided by the Macintosh direct laryngoscope and McGrath video laryngoscope and the intubation time of patients undergoing cesarean section.

**Methods:**

This study was conducted at a private obstetrics and gynecology hospital during one month between June and July 2018. A hundred patients scheduled for elective cesarean section under general anesthesia were randomized into two different group’s as intubated using either McGrath VL or Macintosh DL. The intubation times, Cormack–Lehane grade, percentage of glottic opening, mean arterial blood pressure, and heart rates before and after intubation were compared among the groups.

**Results:**

The McGrath VL significantly reduced the intubation time compared to the Macintosh DL. In the McGrath VL group, better glottic view set the time of tracheal intubation as assessed using the Cormack-Lehane classification system and POGO scores were recorded. After intubation, hemodynamic parameters were significantly higher in the Macintosh DL group than in the McGrath VL group.

**Conclusion:**

The McGrath VL significantly lowered intubation time relative to the Macintosh DL, which may be a critical finding considering the importance of maintaining the mother’s airway for the health of both mother and baby.

## INTRODUCTION

In the United States, preliminary research on anesthesia-related maternal death indicated that more than 52% of maternal mortalities were caused by complications of general anesthesia, largely connected with airway management.^1^ A growing body of research has improved anesthetists’ understanding of airway difficulties in obstetric patients. However, a recent study showed that the incidence of difficulties during intubation and subsequent complications have increased.^2^ In addition, research has suggested that airway difficulties occur eight times more frequently in obstetric patients than in the general population.^3^

In difficult intubation cases, guidelines have provided recommendations for the availability of advanced airway equipment, and neuraxial anesthesia has been encouraged.^4^ A recent publication argued that video laryngoscopy was superior to direct laryngoscopy for the intubation of obstetric patients with normal airways.^5^ Unlike a standard laryngoscope using a Macintosh blade, the McGrath video laryngoscope (VL) results in a glottic view even without bringing into alignment the oral, laryngeal, and pharyngeal axes. The McGrath VL results in a clear picture of the nearby airway anatomy and vocal cords on a liquid crystal display screen mounted on the handle.^6^

In 2633 general anesthesia cases, the incidence of failed intubation was one in 1300, and the incidence of difficult intubation was 4.7%, which is at the lower end of the recorded range of 1.3 to 16.3% in obstetric patients.^7^ Despite emerging evidence that VLs enhance the glottic view and improve intubation success rate, few studies have considered the function of this device, especially when used in obstetric patients.^5^

The primary objective of this study was to test the glottic visualization provided by the Macintosh direct laryngoscope (DL) and McGrath VL and the time taken to intubate patients undergoing cesarean section. The secondary aim of this study was to investigate whether there was a significant difference in hemodynamic parameters as heart rate (HR), and mean arterial pressure (MAP) between patients intubated with either DL or VL. Our hypothesis was that as a result of its improved glottic vision; the McGrath VL would reduce the time taken to intubate patients undergoing cesarean section.

## METHODS

This study was approved by the Istinye University Institutional Review Board (IRB approval number: 2017-KAEK-120/20) and has been registered with the Australian New Zealand Clinical Trial Registry (Trial ID: ACTRN12618000902291). This study was conducted at a tertiary obstetrics and gynecology hospital during one month between June and July 2018. Written informed consents were obtained from all participants both for the interventions and enrollment into the study. A hundred pregnant patients with American Society of Anesthesiologists (ASA) II were recruited to this randomized controlled trial. Patients were aged between 18 and 40 years and were scheduled for elective cesarean section under general anesthesia between June and July 2018. Exclusion criteria were as follows: retrognathia; restricted neck movement; a Mallampati score of IV; emergency surgery; a history of airway-related surgery; renal, hepatic, neuromuscular, or cardiovascular illness; and an American Society of Anesthesiologists (ASA) score of III or IV. Demographic data, including patients’ height, age and weight were recorded. During airway examination, patients’ Mallampati score, thyromental distance, and neck circumference were recorded. Patients were randomized using a sealed-envelope technique. A computer was used to generate random numbers, and patients were randomly allocated to two groups in which they would receive intubation with either the McGrath VL (Aircraft Medical Ltd., Edinburgh, UK) (n=50) or the Macintosh DL (n=50).

Intubation was carried out by attending three anesthesiologists who were certified by the National Society of Anesthesiologists to use VLs and had prior experience of at least 100 successful intubations using VLs. The operating room anesthesiologist was responsible for identifying the laryngoscopy technique to which each randomized patient had been allocated. This was done prior to each patient’s procedure by opening the sealed envelope that had been assigned to the patient. After the patient arrived in the operating room, ASA standard monitoring procedures were applied. All patients received standard general anesthetic regimens, which included intravenous rocuronium bromide (0.6 mg/kg) and intravenous propofol (1 to 2 kg/mg titrations). Intubation time was measured from the point at which the laryngoscope blade was inserted into the mouth, and timing stopped upon the detection of an end-tidal CO_2_ trace. The anesthesiologist rated the glottis visualization by using the Cormack-Lehane classification system and the glottis opening percentage (POGO) while placing the endotracheal tube.^8^ The Cormack-Lehane classification system rates glottic visualization on a 4-point scale, while the POGO score is a percentage. A POGO score of 100% remarked full visualization of the larynx starting from anterior commissure to the posterior cartilage, while 0% indicated a complete absence of glottic opening.

If the specialist anesthesiologist failed to intubate patients in the Macintosh DL group after three trials, then the intubating laryngeal mask airway (LMA) was used to intubate as an emergency precaution. During both VL and DL procedures, a malleable style was inserted into the tracheal tubing in order to guide it. Sevoflurane at the minimum alveolar concentration with an air mixture of 0.6 and fractional determined oxygen of around 0.4 was used to maintain anesthesia. Pressure controlled volume guaranteed mechanical ventilation was used while targeting an end-tidal CO_2_ pressure between 35 and 40 mmHg.

The timing of glottic view, tracheal tube placement, and tracheal intubation were confirmed by CO_2_ waveforms. Mean arterial pressure (MAP) and heart rate (HR) were recorded before intubation and three minutes after intubation.

### Statistical analysis

Number Cruncher Statistical System 2007 and Power Analysis and Sample Size 2008 statistical software (Utah, USA) were used. Descriptive data, including the mean, standard deviation (SD), median, frequency, interquartile range (IQR), rate, and minimum and maximum values, were examined. After evaluating the distribution using a Shapiro-Wilk test, normally distributed data and non-normally distributed data were analyzed using an independent t-test and Mann-Whitney U test, respectively. Normally distributed data were detailed with mean (SD) and not normally distributed data with median [IQR]. A *p* value < 0.05 was considered as statistically significant.

Sample size was calculated based on data from a pilot study that involved 10 patients in each group. In this pilot study, patients in the McGrath VL group had a mean total intubation time that was six seconds shorter and a SD that was 8.7 second shorter than patients in the DL group. To determine a significant difference between intubation times, a two-sided Type-I error of 0.05 and a power of 0.9 were needed for data from 45 patients in each group, assuming an equal standard deviation. We recruited 15% more than this estimate to compensate for dropout, meaning that we invited 105 patients to participate in the study.

## RESULTS

### A total of 100 patients were enrolled in and completed this study (Fig.1).

**Fig.1:**
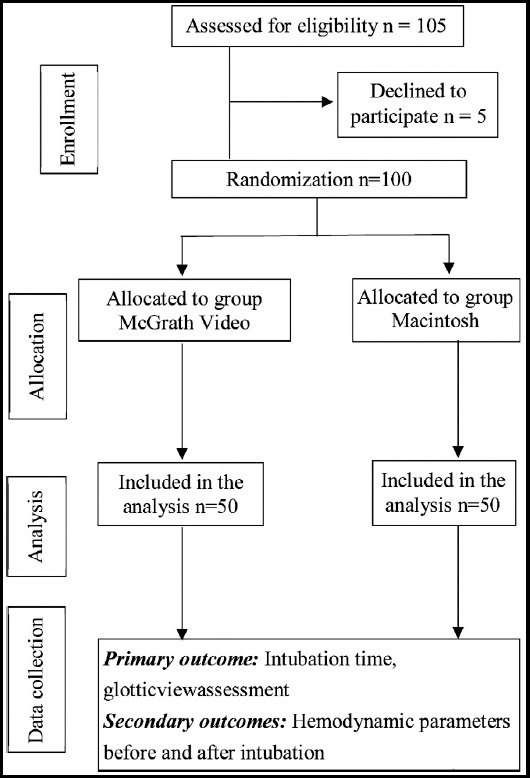
Flow diagram of patients who were enrolled in, excluded from, and completed the protocol.

There were no significant differences in the demographic data and airway evaluations between the two groups (Table-I).

**Table-I:** Demographic data and airway evaluation.

	DL (n = 50)	VL (n = 50)	p
Age	27.5 (24–31)	26 (22–35)	0.65*
Weight (kg)	81.1±6.9	80.3±7.6	0.59^#^
Height (cm)	165.9±6.1	165.6±5.8	0.81^#^
BMI	29.6±2.2	29.3±2.5	0.45^#^
Mallampati	1.86±0.4	1.98±0.6	0.21^#^

# Independent t-test results are expressed as mean ± standard deviation or as numbers of patients.

* Mann-Whitney U-test results are expressed as the median (25^th^–75^th^ interquartile range)

DL: direct laryngoscope, VL: video laryngoscope, BMI: Body Mass Index.

The McGrath VL significantly reduced the time taken to obtain the glottic view compared with the time taken in the Macintosh DL group (p < 0.05). Tracheal tube placement in the Macintosh DL group took significantly longer than in the McGrath VL group (p<0.05). The McGrath VL reduced the time taken to confirm correct placement of the tracheal tube (confirmed by end-tidal CO_2_) compared to the Macintosh DL (p< 0.001, Table-II).

**Table-II:** Time taken for glottic view, tracheal tube placement, and intubation time.

	DL (n =50)	VL (n = 50)	p^#^
Glottic view (s)	19.5±3.9	17.7±4.4	0.028
Tracheal tube placement (s)	32.6±4.7	29.8±5.1	0.007
Intubation time (s)	40.1±5.4	34.7±5.2	<0.001

# Independent t-test results are expressed as mean ± standard deviation

DL: direct laryngoscope, VL: video laryngoscope

In the McGrath VL group, better glottic view set the time of tracheal intubation as assessed using the Cormack-Lehane classification system and POGO scores were recorded compared to those in the Macintosh DL group (Table-III).

**Table-III:** Glottic view assessment.

	DL (n = 50)	VL (n = 50)	p
***Cormack-Lehane grade***			
N (I/II/III/IV)	3/34/9/4	16/32/2/0	0.003^#^
% (I/II/III/IV)	6/68/18/8	32/64/4/0	

POGO (%)	90 (86.75–92)	94.5 (90–96)	< 0.001*

# Independent t-test results are expressed as mean ± standard deviation or as numbers of patients.

* Mann-Whitney U test results are expressed as the median (25^th^–75^th^ interquartile range)

DL: direct laryngoscope, VL: video laryngoscope, POGO: percentage of glottic opening

Baseline MAP and HR were similar between the two study groups. Following tracheal tube insertion, MAP was higher in the Macintosh DL group than in the McGrath VL group. Similarly, after tracheal tube insertion, mean HR was significantly higher in the Macintosh DL group than in the McGrath VL group. In addition, intragroup comparisons of MAP and HR revealed significant increases after intubation (Table-IV).

**Table-IV:** Hemodynamic parameters before blade insertion and 3 minutes after intubation.

	DL (n = 50)	VL (n = 50)	p^#^
***Mean arterial pressure (mmHg)***			
Baseline	79.3±8	77.9±7.4	0.3
Post-intubation	89.6±6.7	82.3±7.5	<0.001
***Heart rate (bpm)***			
Baseline	76.7±7.4	77.6±7.6	0.5
Post-intubation	93.4±8.5	88.7±7.5	0.005

# Independent t-test results are expressed as mean ± standard deviation

DL: direct laryngoscope, VL: video laryngoscope.

## DISCUSSION

In the current prospective randomized controlled study, we hypothesized that McGrath VL may shorten the intubation time of patients undergoing cesarean surgery based on the fact that video laryngoscopes improve the quality of glottic imaging. In our study, the McGrath VL reduced mean intubation time by 5.4 seconds compared to the Macintosh DL. After blade insertion, the time taken to obtain the glottic view and to place the tracheal tube was significantly reduced when using the McGrath VL compared to the Macintosh DL. Higher Cormack-Lehane and POGO scores were achieved using the McGrath VL compared to the Macintosh DL, meaning that video laryngoscopy resulted in better glottic view than direct laryngoscopy in this study.

Anatomical and physiological changes in parturient patients compared to the general population are significant in the practice of obstetric anesthesiology. It has been estimated that women gain 15 to 20 kg during pregnancy.^9^ Enlargement of the breasts during pregnancy may result in difficulties when placing the laryngoscope, particularly in the supine position. Fluid retention in the neck, head, and tissues may reduce the airway in the upper region as well as limiting compliance, which may make laryngoscopy more difficult.^10^ In addition, labor and delivery themselves may result in acute airway variations.^11^,^12^ Upward diaphragm displacement resulting from the expansion of the uterus may impinge on residual and functional capacity, particularly in the supine position.^13^ Efficient airway management in pregnant women promotes the health of both mother and fetus.

Using the McGrath VL resulted in a mean intubation time of 34.7 seconds in the current study. A related study by Arici S et al.^5^ found a longer mean intubation time of 47.25 seconds when using the McGrath VL. Although the primary outcome of Arici S et al.’s study and the current study were similar, Arici S et al. did not report the level of experience of attending anesthesiologists. As such, intubation in the current study may have been carried out by anesthesiologists with more experience, which could account for the shorter intubation time. Taylor AM et al.^14^ recorded a mean intubation time of 35.8 seconds, while Walker L et al.^15^ reported 47 seconds and Shippey B et al.^16^ reported 24.7 seconds. In one study, it took experienced anesthesiologists an average of 98.8 seconds to intubate manikins with manual in-line stabilization by using the McGrath VL.^17^ However, it is not possible to make an explicit comparison between these studies, as the definition of intubation time varied between them. Shippey B et al.^16^ suggested that when using VLs, intubation time may be reduced if the tracheal tube and stylet are adequately prepared.

Generally, the stage of intubation that takes the longest amount of time is the observation of the opening of the glottis. In obese patients undergoing bariatric surgery, Yumul R et al. demonstrated that three VL devices (the McGrath, GlideScope, and Video-Mac) significantly decreased the amount of time taken to view the opening of the glottis relative to a standard DL.^18^ They reported a mean glottic view time of 10 seconds, whereas we found a mean time of 17.7 seconds. In their study of obese patients, they used a 30° ramp to improve glottic view and shorten glottic view time, which may have contributed to their different results. Although our patients underwent an elective surgical procedure, we did not use a 30° ramp, as it was not appropriate to implement this for pregnant patients before the induction of anesthesia.

In the current study, the McGrath VL group exhibited better glottic views at the time of tracheal intubation as evaluated by Cormack-Lehane and POGO scores. Noppens RR et al.^6^ found that the McGrath VL provided better glottic views and quicker intubation times than the Macintosh DL, especially in patients whose Cormack-Lehane score was three or four when using the Macintosh DL. Although we did not use both devices in all patients, airway examinations were similar between the groups, and Cormack-Lehane scores in the McGrath VL group were grade three and below. Various studies have demonstrated the same results, suggesting that glottic view is better when using a VL.^5^,^14^,^16^,^18^ The position of McGrath VL blades relative to the tracheal axis may enhance the implicit view involving the laryngeal inlet, because it is comparatively close to the axis of tracheal opening.^19^

Hypertension is common after tracheal intubation during general anesthesia, and it is usually impacted by many factors, including patient characteristics^20^, opioid treatment during induction^21^, and the form of intubation device used.^22^,^23^ Yokose M et al. concluded that using the McGrath VL may reduce the occurrence of hypertension after tracheal intubation relative to the Macintosh DL.^24^ Successful intubation using the Macintosh DL often requires forcing the arrangement of the pharyngeal and axes in order to establish the glottic view. This maneuver stimulates the supraglottic area and oral tissues, which induces a sympathetic response in the patient. This is generally regarded as the leading cause of excessive hemodynamic response during intubation using a DL.^24^ Application of a VL may reduce the risk of excessive stimulation of the supraglottic region compared to using a DL. A number of studies have asserted that laryngoscopy can increase HR.^25^ Similar to other studies, the results of the current study revealed an increase in MAP and HR during laryngoscopy when using both the Macintosh DL and McGrath VL. After intubation, MAP and HR values were significantly higher in the Macintosh DL group compared to those in the McGrath VL group. Overall, the McGrath VL reduced patients’ hemodynamic response to intubation compared to the Macintosh DL. This may be because the alignment of the pharyngeal and oral axes does not need to be forced when using the McGrath VL.

### Limitations of the study

First, this prospective randomized controlled study could not be conducted in a blinded fashion. Secondly, the anesthesiologist in this study was experienced in airway management and using VLs, which might have influenced the results. This limitation is the result of the fact that obstetric anesthesia was performed only by experienced anesthesiologists in our hospital. Thirdly, the Cormack-Lehane classification system and POGO which were used to assess glottic view during tracheal tube placement can be nominative. Finally, the study population comprised only of selected surgical patients with normal airways; thus, conclusions could be different in patients with difficult airways.

## CONCLUSION

In conclusion, using the McGrath VL for the tracheal intubation of obstetric patients improved the larynx visualization compared to the Macintosh DL. Furthermore, the McGrath VL significantly lowered intubation time relative to the Macintosh DL, which may be a critical finding considering the importance of maintaining the mother’s airway for the health of both mother and baby.

### Author`s Contribution

**MKT** conceived, designed and did statistical analysis & editing of manuscript.

**BA and AGK** did data collection and manuscript writing.

**MKT** did review and final approval of manuscript.
